# Relationship between red cell distribution width and prognosis in patients with breast cancer after operation: a retrospective cohort study

**DOI:** 10.1042/BSR20190740

**Published:** 2019-07-12

**Authors:** Deshun Yao, Zhiwu Wang, Haifeng Cai, Ying Li, Baosheng Li

**Affiliations:** 1 Tianjin Medical University Cancer Institute and Hospital, Tianjian 300060 China; 2 Department of Breast surgery, Tangshan People’s Hospital, Tangshan, Hebei 063000, PR China; 3 Department of Chemoradiotherapy, Tangshan People’s Hospital, Tangshan, Hebei 063000, PR China; 4 Department of Nuclear Medicine, Tangshan People’s Hospital, Tangshan, Hebei 063000, PR China; 5 Department of Radiation Oncology, Shandong Cancer Hospital Affiliated to Shandong University, Shandong Academy of Medical Sciences, Jinan, Shandong 250117, PR China

**Keywords:** breast cancer, prognosis, Red cell distribution width, retrospective study

## Abstract

We retrospectively enrolled 825 breast cancer patients, who was primarily diagnosed in our hospital between January 2009 and December 2014 and explored the relationship between red blood cell distribution width (RDW) and long-term prognosis in patients with breast cancer. There were 412 patients with high RDW (RDW > 13.82) and 413 patients with low RDW (RDW ≤ 13.82). Compared with low RDW group, the high w group has large tumor size (the rate of tumor size >2 cm: 60.7 vs 44.8%, *P*=0.013). The rate of lymph node metastases was higher in the high RDW group thaten that in the low RDW group (62.1 vs 45.8%, *P*=0.000). RDW was positively associated with tumor stage. The high RDW tended to be advanced stage (*P*=0.000). Compared with low RDW group, the high RDW group tended to be higher lymphocyte count (*P*=0.004), elevated fibrinogen (*P*=0.043), and elevated high-sensitivity C-reactive protein (*P*=0.000). The Kaplan–Meier analysis indicated elevated RDW was positively associated with disease-free survival (DFS) (*P*=0.004) and overall survival (OS) (*P*=0.011). The multivariate Cox regression analysis indicated that the high RDW group had poorer OS (Hazard risk [HR] = 2.43; 95% CI: 1.62–3.21; *P*=0.024) and DFS (HR = 1.89; 95% CI: 1.28–3.62; *P*=0.000) compared with low RDW group. The present study found that high pretreatment RDW levels in breast cancer patients were associated with poor OS and DFS. RDW could be a potential predictive factor in differential diagnosis of poor prognosis from all patients.

## Introduction

Breast cancer is one of the most common female malignancies worldwide and with a heavy mortality [1]. The morbidity of breast cancer is prone to be leading female malignancies in China [2]. The metastasis induces local relapse and distant metastasis in breast cancer; therefore, identification of metastasis is of great importance in clinical management and with significant predictive and prognostic value [3]. Although comprehensive treatment based on surgical treatment has significantly improved the prognosis of breast cancer patients, its overall prognosis is still not ideal [4,5]. Based on these concepts, breast cancer should be considered as a systemic disease and some molecular biomarkers should be needed in precision medicine for breast cancer. Therefore, it is of great significance to seek a simple, convenient, and sensitive prognostic indicator of breast cancer for early detection of tumors and guidance of treatment decisions.

Red width cell distribution width (RDW) is an index for evaluating the variation of red blood cell volume. Increased RDW has been treated as a measure of all kinds of anemia such as iron deficiency anemia, vitamin B12 deficiency anemia [6]. Lippi first found that RDW was strongly and independently associated with high-sensitive C-reactive protein (CRP) [7]. Semba et al. also found that the patients with higher RDW level tended to have a higher interleukin-6 level [8]. It is now widely recognized that smoldering inflammation in the tumor microenvironment plays a pivotal role in the initiation, progression, and progression of cancer [9]. Furthermore, previous study also suggested that RDW was associated with poor prognosis in various malignancies [10–13]. Koma reported that elevated RDW predicted poor survival status in patients with lung cancer [14]. In parallel with the present study, Abakay found that RDW was significant predictor of poor prognosis in patients with malignant mesothelioma [15]. In addition, RDW was also related to a higher stage disease according to international staging system and poor prognosis in patients with symptomatic multiple myeloma [16]. Moreover, a previous study indicated that RDW could be a potential biomarker of activity of breast cancer [17]. The data about role of RDW in breast cancer prognosis is scare, especially large sample size. In the present study, we explored the relationship between RDW and long-term prognosis in patients with breast cancer.

## Materials and methods

### Study population

We retrospectively enrolled breast cancer patients who was primary diagnosed in Tanshang People’s Hospital between January 2009 and December 2014. Criteria for inclusion: Patients were newly diagnosed and did not receive any neoadjuvant therapy before surgery administration; Patients belonged to T1-4 N0-3 M0 stage according to the tumor node metastasis classification system (international union against cancer) [18]; all patients were confirmed by pathology tissue. The following patients were excluded: noninvasive breast cancer or stage IV breast cancer or inflammatory breast cancer; patients had received some treatment including chemotherapy or radiotherapy; confirmed diagnostic of pathology was lack; no useful information exaction or follow-up results were obtained. Patients with systemic inflammatory, severe liver and kidney dysfunction, thromboembolic diseases, autoimmune diseases, hepatopathy, diabetes mellitus, hypertension, cardio- and cerebrovascular diseases, serious infection or other tumors were also excluded. All demographic information and pathology were obtained from medical records. The present study was approved by the Ethics Committee of Tangshan People’s Hospital. The research was carried out in accordance with the World Medical Association Declaration of Helsinki, and all subjects provided written informed consent.

### Data collection

We mainly collected the following data from the medical records: age, body mass index (BMI), history of smoking (smoking was defined as current smoking or smoked previously daily), history of diseases. The pathology parameters included tumor size, lymph node metastasis status, distant metastasis, histology grade and adjuvant therapy after the resection. The pathological results were assessed according to AJCC6 criteria by at least two chief physicians. ST >Gallen version 2013 was used to assess the estrogen receptor (ER) positive defined ≥1%; progesterone receptor (PR) positive meant ≥20%; and human epidermal growth factor receptor-2 (HER-2) via immunohistochemical standard [19].

Hematologic testing was conducted on the Beckman Coulter LH-750 Hematology Analyzer (Beckman Coulter, Inc., Fullerton, CA, U.S.A.), automated hematology analyzer, which measures hemoglobin photometrically, including white blood cell counts (WBC), red blood cell, platelet counts, hemoglobin, and RDW. In addition, fasting blood glucose level (FBG), and fasting serum lipid status including triglycerides (TG), triglyceride, low density lipoprotein cholesterol, high density lipoprotein cholesterol (HDL-C), total cholesterol, serum albumin, and CRP levels were also recorded.

The patients were followed up till March 2015. The primary follow-up outcomes were death or recurrence or distant metastatic. Overall survival (OS) was defined as the time from surgery to death, and disease-free survival (DFS) was defined to as the time from surgery to local recurrence or distant metastasis [20].

### Statistical analysis

Study populations were divided into two groups according to the median of RDW (median = 13.82). Continuous data were expressed as mean ± S.D. or median (minimum, maximum) according to the normality of data distribution. The independent sample *t* test or non-parameter test was used between high RDW group (>median) and low RDW group (≤median). The normality test was performed by Kolmogorov–Smirnov method. The category data were expressed using the count and percent. The Chi-square test was used to compare the differences between two groups. The censoring time was defined as the last follow-up time. We used the Kaplan–Meier survival curves with log-rank tests and Cox proportional hazard regression analysis to compare the OS rate and DFS rate, respectively. The multivariate analysis was also used to explore the relationship between RDW and prognosis in patients with breast cancer. All analyses were performed using the SPSS 20.0 and GraphPad Prism 5.0. *P*<0.05 was considered statistically significant.

## Results

### General characteristics of study population

According to the criteria for inclusion and exclusion, 825 patients with breast cancer were included in the final analysis. The mean age was 52.0 ± 9.8. The smoking rate was 8.4–66.1% of patients were lymph node metastasis. The stage I, II, and III were 22.3, 48.1, and 29.6%, respectively. About 89.6% of patients were absent of peritumoral vascular invasion (PVI). The ratios of ER, PR, and Her2 positive were 60.1, 50.7, and 22.4%. About 74.8% of all patients received radical surgery treatment and 25.2% of them received conservative treatment. About 90.0% of patients received chemotherapy. The rates of FEC (5-fu/Farumorubishin/ cyclophosphamide) and TEC (Docetaxel + epirubicin + cyclophosphamide) were 26.1 and 63.9%. The median follow-up time was 47.6 months (range from 5 to 75 months). The study population were divided into two groups (high RDW and low RDW group) according to the median of RDW (RDW = 13.82).

### Relationship between RDW and clinicopathological characteristics

There were 412 patients with high RDW (RDW > median) and 413 patients with low RDW (RDW ≤ median). The Table 1 presented the relationship between RDW and clinicopathological characteristics. Compared with low RDW group, the high RDW group has large tumor size (the rate of tumor size >2 cm: 60.7 vs 44.8%, *P*=0.013). The rate of lymph node metastases was higher in the high RDW group than that in the low RDW group (62.1 vs 45.8%, *P=*0.000). RDW was positively associated with tumor stage. The high RDW tend to be advanced stage (III: 34.2 vs 24.9%; II: 56.1 vs 40.2%; I: 9.7 vs 34.9%. *P=*0.000). The mean age of high RDW group is little higher than that in the low RDW group. But there was no significant difference (*P=*0.460). No significant difference was observed in BMI (*P=*0.425). The smoking rates between two groups were fairly even (8.7 vs 8.0%, *P=*0.778). The rate of PVI absent was 88.3% in the high RDW group and 90.8% in the low RDW group, respectively. There were no significant differences in ER positive rate, PR positive rate, and Her-2 positive rate (*P=*0.599, *P=*0.464, *P=*0.082). Most patients of two groups received radical surgery treatment (74.5 vs 75.1%, *P=*0.857). The rate of chemotherapy after surgery was 91.0% in the high RDW group and 88.9% in the low RDW group. No significant differences were observed in chemotherapy rate and chemotherapy regimens selection (*P=*0.303, *P=*0.080).

**Table 1:** Relationship between RDW and clinicopathological characteristics in patients with breast cancer

Parameters	>Median (*n*=412)	≤Median (*n*=413)	χ^2^/t	*P*-value
Age (y)	52.3 ± 9.3	51.8 ± 10.1	0.740	0.460
BMI (kg/m^2^)	23.6 ± 3.5	23.4 ± 3.7	0.798	0.425
Smoking (n, %)	36 (8.7%)	33 (8.0%)	0.079	0.778
Tumor size, cm			6.110	0.013
≤2	162 (39.3%)	172 (13.2%)		
>2	250 (60.7%)	185 (44.8%)		
Lymph node metastases (n, %)	256 (62.1%)	189 (45.8%)	22.904	0.000
Stage			75.342	0.000
I	40 (9.7%)	144 (34.9%)		
II	231 (56.1%)	166 (40.2%)		
III	141 (34.2%)	103 (24.9%)		
PVI (absent, %)	364 (88.3%)	375 (90.8%)	1.325	0.250
ER positive (n, %)	244 (59.2%)	252 (61.0%)	0.277	0.599
PR positive (n, %)	214 (51.9%)	204 (49.4%)	0.535	0.464
Her-2 positive (n, %)	82 (19.9%)	103 (24.9%)	3.008	0.082
Ki-67>20	236 (57.3%)	232 (56.2%)	0.103	0.748
Type of surgery			0.032	0.857
Radical	307 (74.5%)	310 (75.1%)		
Conservative	105 (25.5%)	103 (24.9%)		
Chemotherapy (n, %)	375 (91.0%)	367 (88.9%)	1.061	0.303
Chemotherapy regimens			5.050	0.080
None	37 (9.0%)	46 (11.1%)		
FEC	121 (29.4%)	94 (22.8%)		
TEC or AC-T	254 (61.6%)	273 (66.1%)		

We also compared the hematology parameters between two groups (Table 2). Compared with low RDW group, the high RDW group tended to have higher lymphocyte count (2.1 ± 0.5 vs 2.0 ± 0.5, *P=*0.004), elevated fibrinogen (3.1 ± 0.8 vs 3.0 ± 0.6, *P=*0.043) and elevated high-sensitivity C-reactive protein (7.5 ± 4.2 vs 4.3 ± 5.6, *P=*0.000). There were no significant differences in red blood cell (*P=*0.102), white blood cell (*P=*0.096), hemoglobin (*P=*0.383), platelet (*P=*0.527), total cholesterol (*P=*0.172), triglyceride (*P=*0.999), HDL-C (*P=*0.649), LDL-C (*P=*0.697), albumin (*P=*0.257), and fasting plasma glucose (*P=*0.076). The Table 2 gives the details.

**Table 2:** Correlations between pre-treatment RDW and clinical hematology parameter in patients with breast cancer

Parameters	>Median	≤Median	t/	*P*-value
Red blood cell, ×10^12^	6.0 ±1.6	6.2 ± 1.9	-1.635	0.102
White blood cell, ×10^9^	8.5 ± 3.5	8.1 ± 3.4	1.665	0.096
Hemoglobin, g/l	131.2 ± 12.5	130.4 ± 13.8	0.873	0.383
Platelet, × 10^9^/l	241.2 ± 62.3	243.8 ± 55.6	-0.632	0.527
Lymphocyte, 10^9^/l	2.1 ± 0.5	2.0 ± 0.5	2.875	0.004
Fibrinogen, g/l	3.1 ± 0.8	3.0 ± 0.6	2.031	0.043
Total cholesterol, mmol/l	1.3 ± 0.8	1.4 ± 1.1	-1.366	0.172
Triglyceride, mmol/l	1.3 ± 1.1	1.3 ± 1.0	0.000	0.999
HDL-C, mmol/l	1.38 ± 0.32	1.37 ± 0.31	0.456	0.649
LDL-C, mmol/l	3.1 ± 0.67	3.1 ± 0.8	0.389	0.697
High-sensitivity C-reactive protein, mg/l	7.5 ± 4.2	4.3 ± 5.6	9.283	0.000
Albumin, g/l	25.4 ± 10.4	26.3 ± 12.3	-1.135	0.257
Fasting plasma glucose, mmol/l	6.1 ± 2.1	5.9 ± 0.9	1.779	0.076

### Cox regression of DFS and OS

**Figure 1:**
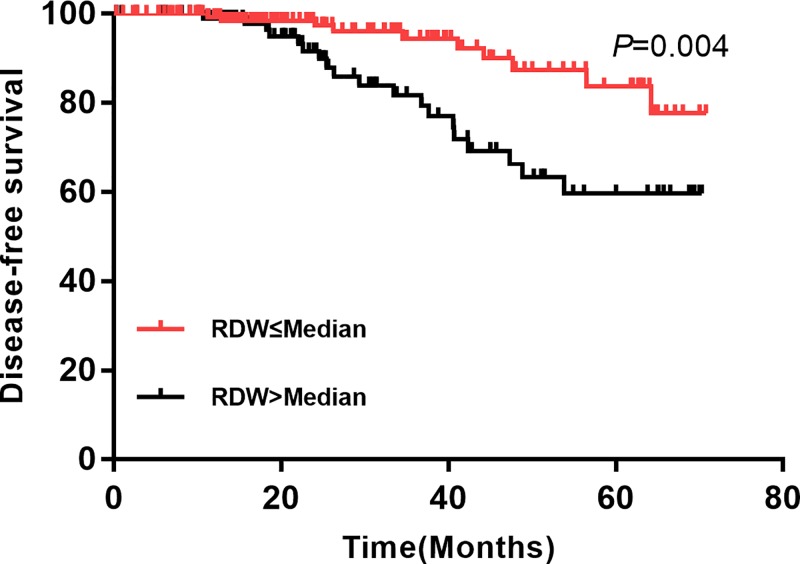
Comparison of OS between high RDW and low RDW group in patients with breast cancer

We performed univariate (Table 3) multivariate (Table 4) cox regression analysis for DFS and OS, respectively. The univariate cox regression indicated elevated RDW was positively associated with local recurrence/distant metastasis (HR = 2.89; 95% CI: 2.14–5.34; *P=*0.000, Figure 1). In addition, age >50 (HR = 1.04; 95% CI: 1.01–2.43), stage III (HR = 1.65; 95% CI: 1.23–3.18), ER positive (HR = 0.34; 95% CI: 0.12–0.76), PR positive (HR = 0.41; 95% CI: 0.37–0.88), PVI present (HR = 5.12, 95% CI: 2.37–8.97), elevated lymphocyte (HR = 0.46; 95% CI: 0.38–0.79) and high-sensitivity CRP (HR = 2.12; 95% CI: 1.56–3.86) were also related to local recurrence/distant metastasis. The multivariate cox regression suggested that RDW was an independent predictor for local recurrence/distant metastasis (HR = 1.89; 95% CI: 1.28–3.62, *P=*0.000). otherwise, stage III, PVI present and high-sensitive CRP also increased the risk of recurrence and metastasis.

**Table 3:** Univariable cox regression analysis of DFS and OS for patients with breast cancer

Parameter	Disease-free survival		Overall survival	
	HR (95% CI)	*P*-value	HR (95% CI)	*P*-value
Age (>50 vs ≤50)	1.04 (1.01–2.43)	0.037	1.17 (1.31–3.28)	0.017
BMI ≥ 24	1.34 (0.67–3.16)	0.165	1.47 (0.59–3.48)	0.276
Smoking (yes vs no)	1.75 (0.87–2.52)	0.431	1.68 (0.48–3.01)	0.591
Tumor size > 2 cm	2.01 (0.41–3.61)	0.529	2.13 (0.18–3.98)	0.618
Stage				
II vs I	1.34 (0.62–2.64)	0.912	1.47 (0.59–2.73)	0.889
III vs I	1.65 (1.23–3.18)	0.024	1.86 (1.42–2.95)	0.013
PVI present	5.12 (2.37–8.97)	0.000	4.18 (2.68–9.07)	0.000
ER positive	0.34 (0.12–0.76)	0.031	0.26 (0.18–0.66)	0.015
PR positive	0.41 (0.37–0.88)	0.022	0.39 (0.26–0.76)	0.034
HER2 positive	0.46 (0.28–2.94)	0.067	0.51 (0.35–2.86)	0.102
Ki-67	1.34 (0.68–3.57)	0.135	1.71 (0.58–4.05)	0.345
Conservative surgery	0.64 (0.34–4.12)	0.413	0.71 (0.28–5.36)	0.552
Chemotherapy	1.37 (0.48–2.64)	0.841	1.64 (0.72–3.81)	0.863
Red blood cell, ×10^12^	0.61 (0.37–1.46)	0.226	0.58 (0.29–2.18)	0.336
White blood cell, ×10^9^	0.54 (0.34–2.41)	0.161	0.82 (0.45–3.67)	0.362
Hemoglobin, g/l	0.43 (0.18–2.38)	0.257	0.68 (0.28–3.41)	0.368
Platelet, n	1.72 (1.23–3.57)	0.044	2.13 (1.22–3.23)	0.025
Lymphocyte, n	0.46 (0.38–0.79)	0.023	0.46 (0.29–1.08)	0.051
Fibrinogen, g/l	1.01 (0.64–2.34)	0.501	1.17 (0.53–3.68)	0.501
Total cholesterol, mmol/l	1.12 (0.85–3.67)	0.537	0.89 (0.76–2.37)	0.628
Triglyceride, mmol/l	2.01 (0.82–4.61)	0.635	1.89 (0.88–3.96)	0.729
HDL-C, mmol/l	0.35 (0.21–2.49)	0.354	0.46 (0.35–3.08)	0.429
LDL-C, mmol/l	1.63 (0.52–3.10)	0.789	1.85 (0.63–3.58)	0.754
High-sensitivity C-reactive protein, mg/l	2.12 (1.56–3.86)	0.000	2.37 (1.18–4.26)	0.000
Albumin, g/l	1.59 (0.67–4.18)	0.801	1.76 (0.85–4.57)	0.836
Fasting plasma glucose, mmol/l	1.26 (0.91–2.68)	0.734	1.87 (0.76–3.14)	0.693
RDW, %>median	2.89 (2.14–5.34)	0.000	2.76 (1.84–4.37)	0.000

**Table 4:** Multivariable cox regression analysis of DFS and OS for patients with breast cancer

Parameter	Disease-free survival		Overall survival	
	HR (95% CI)	*P*-value	HR (95% CI)	*P-*value
Stage				
II vs I	1.37 (0.34–5.67)	0.790	1.94 (0.98-3.83)	0.354
III vs II	2.21 (1.64–3.69)	0.015	1.70 (1.32–2.64)	0.025
PVI present	4.78 (2.28–7.65)	0.000	5.34 (3.41–9.36)	0.001
PR positive	0.36 (0.28–0.73)	0.018	0.41 (0.17–0.86)	0.023
Platelet, n	-	-	1.85 (1.58–3.67)	0.034
High-sensitivity C-reactive protein, mg/l	1.87 (1.29–4.61)	0.003	2.15 (1.67–5.31)	0.014
RDW, %	1.89 (1.28–3.62)	0.000	2.43 (1.62–3.21)	0.024

**Figure 2:**
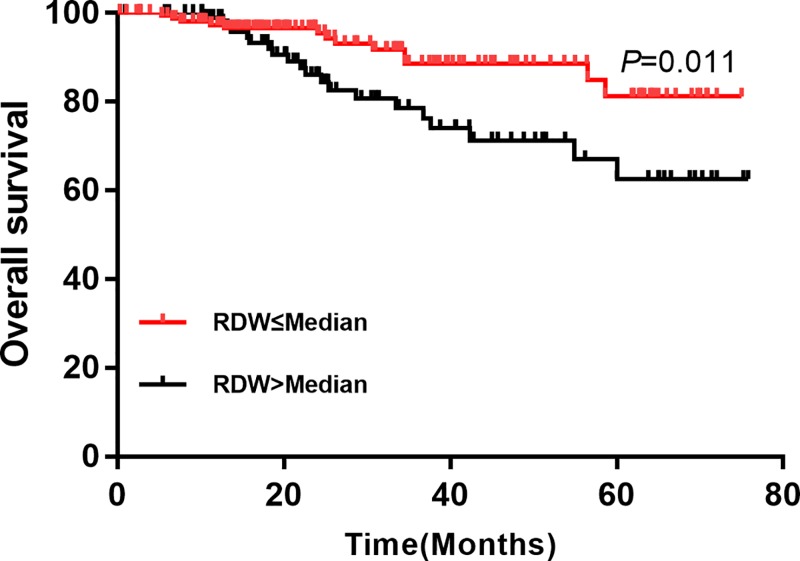
Comparison of DFS between high RDW and low RDW group in patients with breast cancer

For OS, the univariate that the OS rate of high RDW was significantly lower than that in the low RDW group (HR = 2.76; 95% CI: 1.84–4.37; *P=*0.000, Figure 2). Other relative factors included age >50, stage III, ER positive, PR positive, platelet, and high-sensitivity CRP. The multivariate analysis indicated that the high RDW group had poorer prognosis compared with low RDW group (HR = 2.43; 95% CI: 1.62–3.21, *P=*0.024). We also found that reduced platelet was also positively associated with poor prognosis in patients with breast cancer (HR = 1.85; 95% CI: 1.58–3.67; *P=*0.000). Besides, stage III, PVI present and elevated high-sensitivity CRP were also associated with poor prognosis in patients with breast cancer (*P*<0.05). The details were presented in the Table 4.

## Discussion

The present study suggested that elevated preoperative RDW was associated with poor prognosis in patients with breast cancer and was an independent predictor of poor survival in women with breast cancer. Our results indicated that we should pay more attention on these patients who have higher preoperational RDW. Also, considering that RDW is an easily available and inexpensive parameter in blood routine examination. The RDW could be a new accurate and reproducible detected index to distinguish breast cancer patients with poorer prognosis. Previous study also explored the relationship between red cell distribution width and prognosis in patients with breast cancer [21]. There are several different points. First, the population setting is different from previous study. The previous was limited in young breast cancer patients with age under 40 years old, while the present study including patients whose age was more than 18 years old. Second, the sample size is different. The present study has larger sample size including 825 patients with breast cancer that are four-times as many as previous study (*n*=203). Third, previous did not report the clinical hematology parameters, and the present study provided this important information such as high-sensitivity CRP. The present study gives more accurate estimation about the role of RDW in the prognosis of patients with breast cancer.

As a routinely available index of the systemic inflammatory response, RDW has been suggested to be associated with adverse outcomes in different cancer patients setting.

Warwick et al. investigate the association of RDW in patients undergoing lung resections for non-small-cell lung cancer with respect to in-hospital morbidity, mortality, and long-term survival. The present study is a retrospective study with a total of 917 patients with no-small-cell lung cancer. They found RDW is a significant factor after risk adjustment, determining in-hospital morbidity, mortality and long-term survival in patients post-potentially curative resections for non-small-cell lung cancer [22]. Albayrak et al. investigated the utility of RDW as a simple and readily available marker in prostate cancer, as well as to evaluate RDW as a predictor of progression in prostate cancer patients [23]. They reported that the mean RDW value of prostate cancer patients was 14.6, compared with 13.7 in the healthy control group (*P=*0.001). A higher RDW was associated with an increased risk of progression, whereas a lower RDW value was correlated with a low risk of progression. But this is a study with small sample size including 62 newly diagnosed patients and 62 healthy controls. Further research is needed for these patients. In parallel with two studies above, Kust et al. assessed clinical and prognostic value of RDW in patients with colorectal cancer. They reported that elevations in both pre- and postoperative RDW values had significant effects on survival in univariate and multivariate analyses. Effects were found to be independent of tumor related features, stage of the disease, development of anemia, and aberrant inflammatory response to tumor [24]. The RDW not only had adverse influences on long-term outcomes, but even short-term outcomes. Yazici et al. investigate the role of red cell distribution width in predicting prognosis in gastric cancer patients. They found that preoperative RDW levels were significantly higher in patients with short-term mortality (17.9 +/- 4.3 vs 16 +/- 3.2, *P=*0.015). In high RDW group, the incidence of advanced gastric cancer was significantly higher (75 vs 51%, *P=*0.002), whereas DFS (0.035) and OS (*P=*0.04) were lower. These results indicated that the frequency of advanced cancer was high in patients with high RDW values. High RDW values were strongly associated with short-term mortality [25]. Our data supported the role of RDW in the poor prognosis for breast cancer patients. These studies suggested that RDW is an important biomarker in cancers.

The mechanism that could explain the relation between RDW and survival or disease activity is not clear, but it is considered that high RDW is caused by chronic inflammation, poor nutritional status, oxidative stress, and age-related diseases that lead to changes in erythropoiesis [26,27]. As we all know that inflammation in the rumor microenvironment promotes tumor growth, invasion, angiogenesis, and eventually metastasis. Elevated inflammatory markers such as CRP, neutrophil to lymphocyte ratio, interleukin-6, have been related to poorer survival amongst breast cancer patients [28–30]. The inflammation leaded to changes in red blood cell maturation by disturbing the red cell membrane, inducing increased RDW. This may be one of the mechanisms. Our study found that higher RDW values, with larger primary tumor was significantly associated with the number of axillary lymph node metastasis and the late stage, a reasonable explanation is a malignant tumor may extend the inflammatory response in the process of its progress time and increases the circulation cytokine levels, RDW may be potential biomarkers of cancer growth and metastasis activity. Another reason could be oxidative stress. Both endogenous and exogenous sources of reactive oxygen species result in increased oxidative stress in the cell. Excess reactive oxygen fumed can result in damage to and modification of cellular macromolecules most importantly genomic DNA that can produce mutations [31]. In addition, oxidative stress modulates gene expression of downstream targets involved in DNA repair, cell proliferation, and antioxidants. The modulation of gene expression by oxidative stress occurs in part through activation or inhibition of transcription factors and second messengers. The role of single nuclear polymorphism for oxidative DNA repair and enzymatic antioxidants are important in determining potential human cancer risk [32]. Previous study reported that elevated red cell distribution width level is associated with oxidative stress and inflammation in a canine model of rapid atrial pacing [33]. Just like inflammation, the status of oxidative stress may reduce RBC survival and lead to elevated levels of RDW [34].

The main strength of our study is the large sample size and data is collected pretreatment, which excluded some potential confounding factors. One of the limitations is that this is a retrospective and single center study and we did not explore the molecular mechanisms. The other limitation is that the selection bias may exist for retrospective study. Some study population were ignored because data were missing. Some factors may be imbalance between two groups. These factors cannot be available. Further research is required.

In conclusion, the present study found that high pretreatment RDW levels in breast cancer patients was associated with poor OS and DFS. RDW could be a potential predictive factor in differential diagnosis of poor prognosis from all patients. Future studies should explore the specific molecular mechanism and focussed on long-term outcome. Patients may benefit from regular clinical surveillance for RDW.

## Abbreviations

BMI: body mass index
CRP: C-reactive protein
DFS: disease-free survival
ER: estrogen receptor
FBG: fasting blood glucose level
HDL-C: high density lipoprotein cholesterol
HR: hazard risk
LDH-C: low density lipoprotein cholesterol
OS: overall survival
PR: progesterone receptor
RDW: red blood cell distribution width
TG: triglycerides
WBC: white blood cell
